# Performance of a Two-Week Rehabilitation Improves Motor Function in Inpatients with Progressive Supranuclear Palsy: A Pre–Post Study

**DOI:** 10.3390/brainsci15010088

**Published:** 2025-01-17

**Authors:** Naomi Matsuda, Yasuyuki Takamatsu, Makoto Sawada, Ikuko Aiba

**Affiliations:** 1 Department of Rehabilitation, NHO Higashinagoya National Hospital, Nagoya 465-8620, Japan; naomatsu1120@gmail.com; 2 Department of Rehabilitation Sciences, Graduate School of Life and Health Sciences, Chubu University, Kasugai 487-8501, Japan; 3 Department of Physical Therapy, College of Life and Health Sciences, Chubu University, Kasugai 487-8501, Japan; y_takamatsu@isc.chubu.ac.jp; 4 School of Physical Therapy, Faculty of Rehabilitation, Reiwa Health Sciences University, Fukuoka 811-0213, Japan; m.sawada@kyoju.ac.jp; 5 Department of Neurology, NHO Higashinagoya National Hospital, Nagoya 465-8620, Japan

**Keywords:** progressive supranuclear palsy, Richardson’s syndrome, progressive gait freezing, rehabilitation, short-term, balance, gait

## Abstract

**Background**: Progressive supranuclear palsy (PSP) is characterized by early postural instability and gait dysfunction, with frequent falls. Rehabilitation is an important therapeutic approach for motor dysfunction in patients with PSP. However, no conclusions have yet been drawn regarding the beneficial effects of rehabilitation in PSP, including the optimal duration of rehabilitation and differences in treatment effects among PSP subtypes. Herein, we investigated the effects of short-term rehabilitation and separately analyzed the effects on patients with PSP-Richardson’s syndrome (RS) and PSP-progressive gait freezing (PGF). **Methods**: The participants underwent several therapeutic exercise programs individualized for each participant, performed over 2 weeks. Analysis was performed on 25 patients with PSP-RS and eight with PSP-PGF. **Results**: Short-term rehabilitation improved the Berg Balance Scale score in both the PSP-RS and PSP-PGF groups, step length on the symptom-dominant side in PSP-RS, the coefficient of variation of step length on the symptom-dominant side, and the stance phase of the Symmetry Index in PSP-PGF. **Conclusions**: Overall, this 2-week short-term rehabilitation intervention was shown to have beneficial effects on balance in patients with PSP-RS and PSP-PGF.

## 1. Introduction

Progressive supranuclear palsy (PSP) is a progressive neurodegenerative disorder characterized by the deposition of tau proteins in neurons of the basal ganglia resulting in Parkinsonian-like symptoms [1,2]. PSP has the typical Richardson’s syndrome (RS) and the other clinical subtypes of progressive gait freezing (PGF), etc., and they are characterized by a wide variety of symptoms [3]. The key clinical features of PSP-RS include ocular motor dysfunction, postural instability, and cognitive dysfunction. Early onset of postural instability and gait dysfunction induces frequent falls, often resulting in fractures or other injuries [4,5]. In contrast, the clinical features of one of the subtypes of PSP-PGF include progressive onset of gait disturbance with initial hesitation and subsequent freezing of gait without rigidity, tremor, dementia, or eye movement abnormalities during the first three years of the disease [3]. The prevalence of PSP-RS and PSP-PGF combined per 100,000 population is 17.90, whereas that of PSP-RS is only 14.32 [6]. PSP-PGF is characterized by a lower prevalence and longer duration of disease compared to PSP-RS. In both subtypes, the symptom progression reduces the activities of daily living (ADL) and quality of life (QoL). Currently, there is no pharmacological treatment for PSP, and the treatment remains symptomatic. Consequently, rehabilitation is an important therapeutic approach for motor dysfunction in PSP. Rehabilitation exercises affect the muscles and nervous system, improving motor performance, such as muscle strength, balance, and walking ability. For Parkinson’s disease (PD), evidence suggests that exercise is beneficial in reducing the severity of motor symptoms and improving functional mobility, balance, health-related QoL, muscle strength, and gait speed [7,8]. However, no conclusive evidence has been found regarding the benefits of exercise for PSP [9].

We previously reported that a four-week multiple therapeutic exercise program exerted beneficial effects on balance function among patients with PSP [10]. Other intervention studies on balance training [11], body-weight-supported treadmill training [12], auditory-visual cue feedback [13,14], and multidisciplinary intensive rehabilitation treatments [14] have also investigated their effectiveness at improving gait and balance function. Most of these studies had an intervention period of 4 to 8 weeks [9]. Although the frequency of rehabilitation varied from week to week across studies, rehabilitation is considered beneficial to the patient if it can be effective within a shorter period, as long-term interventions can lead to a decline in QoL and can be both physically and financially burdensome. Only one single case study showed that backward gait training combined with gait-synchronized transcranial alternating current stimulation improved balance function in a short-term intervention (4 min × four sessions), although this study investigated the effect of a long-term intervention (5 weeks) [15]. In PD, the benefits of multidisciplinary short-term rehabilitation on physical function and QoL have been investigated [16,17,18,19]. However, the period of rehabilitation intervention for PSP is mostly 4–8 weeks, and there are no reported studies on the effectiveness of short-term rehabilitation.

Furthermore, several studies have investigated typical PSP- RS; however, there is only one case study of atypical PSP [20]. Therefore, no conclusion regarding the beneficial effects of rehabilitation for patients with PSP has yet been established in the literature [9,21,22], including the optimal rehabilitation periods and differences in the effects by subtype.

To address this limited knowledge, this study aimed to investigate whether a 2-week short-term intensive rehabilitation program improves balance function, motor function, and gait function in patients with PSP.

## 2. Materials and Methods

### 2.1. Participants

This retrospective pre–post study enrolled patients with PSP at the National Hospital Organization Higashinagoya National Hospital between September 2016 and March 2024. The inclusion criteria were patients with probable PSP-RS and PSP-PGF, according to the Movement Disorder Society criteria for the clinical diagnosis of PSP [3]. The exclusion criteria were as follows: (1) a modified Rankin Scale (mRS) score ≥5, (2) patients with subtypes except PSP-RS and PSP-PGF, (3) patients who had previously undergone rehabilitation treatment in the hospital, (4) inability to walk without assistance for at least 10 m, (5) patients discharged within 2 weeks, and (6) patients with missing values in the outcome data.

Patients were hospitalized for rehabilitation. The medications of all participants remained unchanged to maintain a stable condition during the rehabilitation period.

### 2.2. Measured Outcomes

The age, disease duration, sex, subtype, frontal assessment battery, mini-mental state examination, mRS, and PSP rating scale (PSPRS) [23] scores of all patients were evaluated. All patients were evaluated for balance, motor function, and gait function pre and post two weeks of rehabilitation. Balance and basic motor functions were evaluated using the Berg Balance Scale (BBS) [24], pull test [23,25,26], timed up and go test (TUG) [27], 10 m Walk Test (maximum speed) [28], 2 min walking test (2 MWT) [29,30], and knee extensor strength [31]. Gait function was evaluated using a gait analysis device. All patients were evaluated for balance and basic motor functions pre and post 2 weeks rehabilitation. Participants underwent the following tests:

(1) PSPRS [23]: The PSPRS was developed to assess disease severity in patients with PSP. Furthermore, PSPRS assesses characteristic symptoms associated with PSP, including behavioral change, ocular-motor, gait, and balance disfunctions. The maximum total score is 100 points. Higher scores indicate high disease severity. The PSPRS subitem scores and total score were evaluated as baseline, and the scores of V: limb movements and VI: gait and midline were evaluated pre- and post-interventions.
(2) Pull test [23,25,26]: The pull test is used for evaluating postural stability (0–4 points) as a component of PSPRS. The examiner stands behind the patient and applies a strong pull on the shoulders with the patient erect with eyes open and feet comfortably apart.
(3) BBS [24]: This evaluated the individual’s balance abilities during the performance of 14 items (0–4 points per item), such as sitting, standing, and one leg standing, and positional changes. The maximum total score is 56 points. Higher scores indicate good balance ability.
(4) TUG [27]: This evaluation consisted of the participant standing up from a sitting position in the chair with a seat height of 40 cm, walking a distance of 3 m, then passing around a cone, returning, and sitting back down in the chair. To assess maximum walking speed, they were asked to walk as fast as possible without running.
(5) The 10 m Walk Test [28]: For the 10 m Walk Test, two end lines and two buffer lines were taped on the ground. Each end line was 14 m from the other and the buffer each line was 2 m from the end line. The buffer lines controlled for acceleration and deceleration. The time to walk the middle 10 m was recorded using a stopwatch. To assess maximum walking speed, they were asked to walk as fast as possible without running.
(6) 2 MWT [29,30]: The 2 MWT was performed indoors, along a long, flat, straight, enclosed corridor with a hard surface. The walking course was 30 m in length, and the turnaround point was marked with a cone (such as an orange traffic cone).
(7) Knee extensor strength [31]: Isometric knee extension muscle strength was measured using handheld dynamometers (HHDs, μTas F-1; Anima Corp., Tokyo, Japan). During the measurements, the participants sat on a bench and adjusted the position of their gluteal region such that the bench leg was behind the lower extremity on the measurement side. The bench was adjusted to a height at which both feet of the participant were off the floor. The HHD sensor was placed over the front surface of the distal part of the lower extremity, and the lower edge of the sensor was fixed using Velcro at the height of the upper edge of the medial malleolus. The measurement leg on which the sensor was applied and the bench leg directly behind the measurement leg was tied and fixed using a belt. The participants measured isometric knee muscle extension strength at maximal effort for 5 s while the knee joint was at a flexion angle of 90°, kept their trunk straight, and crossed their arms in front of the chest. For representative values, the values (kgf) measured using the HHD were divided by the body weight (kg).
(8) Gait parameters [32,33]: Gait parameters were measured using a thin-type sensor sheet (Sheet-Type Gait Analyzer WalkWay MW-1000; Anima Corp., Tokyo, Japan) that detected foot pressure using a displacement sensor. Participants walked 6.4 m at their most comfortable speed, which was the distance of the test, plus the additional path of 2 m anterior and posterior to the entire length of the 2.4 m device. Three measurements were obtained and the average values were calculated. The parameters measured included the gait cycle time (s), stance phase time (s), swing phase time (s), step length (cm), and step width (cm). Furthermore, the coefficient of variation (CV) of these parameters were evaluated. Further, the gait parameters from the device were used to calculate the different indices of gait symmetry. The symmetry indices (SIs) between the dominant and non-dominant sides in the gait cycle, stance phase, swing phase, step length, and step width were calculated using the following equation: SI = [2 (symptom-dominant − symptom non-dominant)/(symptom-dominant + symptom non-dominant)] × 100 (%) [34,35].

All patients were evaluated for balance, motor function, and gait function pre and post two weeks of rehabilitation.

### 2.3. Intervention Programs

The intervention was performed for 60–80 min per day, 5 days per week, for 2 weeks, and the intervention program was multiple therapeutic exercise programs, including muscle strength training, gait exercises, and ADL training, with a focus on balance training (Appendix A) [10]. Balance training was performed to improve postural stability and control and included exercises such as holding and standing stable or unstable, reaching, stepping, and turning. Muscle strength training focused on core training and performing leg raises, bridges, crunches, squats, heel and toe raises, front lunges, side lunges, etc. Gait training focuses on balancing the gait; as a result, the patient maintains postural stability while walking and performs tandem walking, backward walking, side walking, and treadmill gait training. In addition, training on walking safely with a cane or walking aid was provided. ADL training included changing and holding postures, dressing, using the toilet, walking indoors and outdoors, and bathing. The program was customized for each patient by physical and occupational therapists and was performed under the guidance of a therapist.

### 2.4. Statistical Analysis

The sample size was determined based on previous studies on PSP rehabilitation interventions [10,11,14]. The normality of the distribution of all variables was evaluated using the Shapiro–Wilk test. The Wilcoxon signed-rank or paired *t*-tests were used to compare pre- and post-rehabilitation data. In addition, the effect size (ES, r) was calculated using the test statistics. To interpret the resulting number, we followed this general guide developed by Cohen, in which ES values < 0.1, 0.1–0.3, 0.3–0.5, and >0.5 indicate is trivial, small, moderate, and large effects, respectively [36,37]. The ES is a statistical measure that quantifies the magnitude of an observed effect. Unlike statistical significance, which indicates only whether an effect exists, ES provides information on the practical or clinical importance of a result.

Data were reported as the mean ± standard deviation for normally distributed data and number for discrete variables. Statistical analyses were performed using SPSS software version 24.0 (IBM Inc., Armonk, NY, USA). Statistical significance was set at *p* < 0.05. 

## 3. Results

### 3.1. Analysis Subject and the Clinical Characteristics

**Figure 1:**
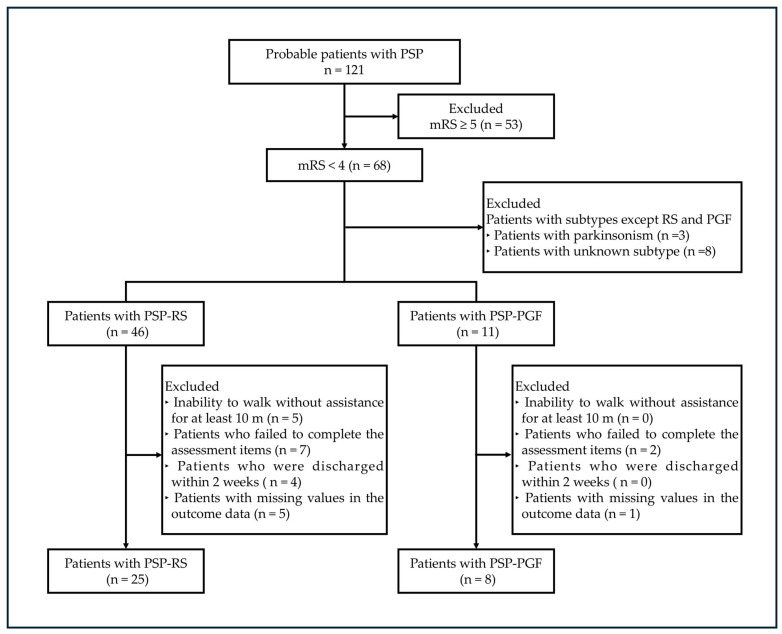
Flow chart of study population selection.

A total of 121 patients with probable PSP were enrolled in this study. Patients were excluded for the following reasons: (1) a score of mRS ≥ 5, *n* = 53; (2) subtypes except RS and PGF, *n* = 11; (3) inability to walk without assistance for at least 10 m, *n* = 5; (4) failed to complete the assessment items, *n* = 9; (5) discharged within 2 weeks, *n* = 4; and (6) missing values in the outcome data, *n* = 6. Consequently, the analysis was performed [11] on 25 patients with PSP-RS and eight patients with PSP-PGF (Figure 1). 

Table 1 lists the demographic and clinical characteristics of the participants who underwent rehabilitation at the hospital. Only the duration of disease was longer in patients with PGF than RS (RS: 41.3 ± 28.5 months, PGF: 77.3 ± 35.4 months, *p* = 0.006); otherwise, their characters were comparable at demographic and clinical characteristics.

### 3.2. Effect on Balance and Motor Function Outcomes

Table 2 presents the comparisons and ES of balance and motor function pre- and post-rehabilitation in patients with PSP-RS and PSP-PGF. In PSP-RS, a significant difference was found in the BBS score (pre: 40.4 ± 8.7 score, post: 43.9 ± 6.7 score, *p* = 0.002), and the ES was large (r = 0.57). No significant difference was found in the knee extensor strength–weight ratio on symptom non-dominant side (pre: 36.0 ± 9.4 kgf/kg, post: 38.5 ± 10.0 kgf/kg, *p* = 0.093), but the ES was large (r = 0.57). No significant difference was found in the 10 MWT (pre: 73.1 ± 17.5 m/min, post: 68.5 ± 14.1, *p* = 0.096), but the ES was moderate (r = 0.33). The 10 MWT of post-rehabilitation was slower than that of pre-test. No significant differences were shown in the other outcomes, and the ES scores were trivial, small, or no effect. In PSP-PGF, significant differences were found in the BBS score (pre: 43.0 ± 7.7 score, post: 48.1 ± 5.4 score, *p* = 0.002) and the ES was moderate (r = 0.48). No significant difference was found in the TUG (pre: 15.3 ± 7.0 s, post: 12.1 ± 3.2 s, *p* = 0.093), but the ES was moderate (r = 0.34). In the other outcomes, no significant differences were shown and the ES values were trivial, small, or had no effect.

### 3.3. Effect on Gait Function Outcomes

Table 3 presents the comparisons and ES of gait function pre- and post-rehabilitation in PSP-RS and PSP-PGF. In patients with PSP-RS, a significant difference was found in the step length on the symptom-dominant side (pre: 42.5 ± 7.8 cm, post: 45.3 ± 6.1 cm, *p* = 0.046), while the ES was moderate (r = 0.39). The following parameters were not found to have any significant difference, but the ES values were moderate: step length on the symptom non-dominant side (pre: 42.7 ± 8.6, post: 45.5 ± 9.1 cm, *p* = 0.053, r = 0.38), CV of the gait cycle on the symptom-dominant side (pre: 4.4 ± 2.5, post: 5.2 ± 2.3, *p* = 0.097, r = 0.33), and CV of the stance phase (pre: 5.5 ± 2.7, post: 6.7 ± 3.7, *p* = 0.079, r = 0.35). The CV of gait cycle and stance phase on the symptom-dominant side were larger post than pre. In the other outcomes, no significant differences were shown and these ES were trivial, small or no effect.

In patients with PSP-PGF, the following parameters were found to have significant differences, with a moderate ES; CV of the step length on the symptom-dominant side (pre: 8.8 ± 3.4, post: 5.3 ± 2.2, *p* = 0.036, r = 0.42) and the stance phase of the SI (pre: 6.1 ± 4.2, post: 2.7 ± 2.2, *p* = 0.025, r = 0.45). No significant differences were found in the step length on the symptom non-dominant side (pre: 8.9 ± 4.1, post: 6.3 ± 3.2, *p* = 0.068), but the ES was moderate (r = 0.37). For the other outcomes, no significant differences were observed, and the ES were trivial, small, or had no effect.

## 4. Discussion

**Figure 2:**
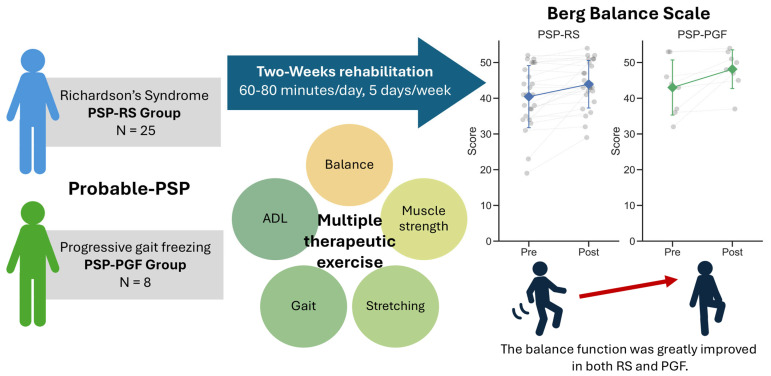
Outline drawing of the current study.

Herein, we investigated the effects of a 2-week short-term rehabilitation intervention on motor function in patients with PSP-RS and PSP-PGF, finding that intervention improved the balance function in both subtypes (Figure 2). Because the type and dosage of medication remained unchanged during the study period, the results of this study can be considered attributable to rehabilitation. Furthermore, this is the first study to show the effect of rehabilitation on PSP-PGF, whereas the majority of reports have focused on PSP-RS.

Prior research has reported that balance training, gait training with a body-weight-supported treadmill, or robot-assisted gait training improve balance function in PSP [12,13,14,15,38,39,40]. We previously reported that balance function, particularly the BBS score and pull test, was improved by a multiple therapeutic exercise program for 4 weeks in patients with typical PSP-RS [10]. In this study, the same program induced a significant improvement and moderate effect on the BBS score just for 2 weeks in not only PSP-RS and PSP-PGF. However, no improvements in the pull test were observed in this study. Therefore, a 2-week intervention with this program would be sufficiently effective for comprehensive balance function, but not for postural instability, in patients with PSP. Alternatively, as postural instability is also one of the major motor symptoms in PSP, with severe consequences for patients, the intervention period of 2 weeks might have been too short to bring about an improvement. The BBS has previously been shown to predict falls in patients with PD and neurodegenerative diseases [41,42]. Therefore, short-term interventions to improve the overall balance function may help prevent falls in patients with PSP.

In the current study, although moderate-to-high ES was observed in the gait speed of the 10 MWT (decline), knee muscle strength of the non-dominant leg (improvement) in the PSP-RS group, and TUG time (improvement) in the PSP-PGF group, we observed no significant improvements in motor function (TUG, 10 MWT, 2 MWT, and knee extensor strength), except for BBS. It has previously been reported that rehabilitation, such as body-weight-supported treadmill gait training [12] and robot-assisted walking [39], improved gait speed in PSP. The program in this study was designed to improve balance function, and strength training was performed with body weight, likely resulting in an insufficient load to improve muscle strength. Furthermore, walking exercises, such as side walking and tandem walking, emphasize maintaining posture while walking rather than targeting improvements in walking speed. Therefore, to improve other motor performances, such as muscle strength and walking speed, it is necessary to implement a program specifically tailored to these goals. Furthermore, fall risk cutoff scores for gait speed and TUG suggest that the higher the speed, the lower the risk of falling in patients with PD and in the elderly [43,44,45,46]. However, patients with PSP develop postural instability from an early onset and have difficulty controlling their balance, resulting in gait instability and repeated falls. For these reasons, the focus should be on improving gait stability rather than gait speed because patients with PSP experience repeated falls due to postural instability from an early stage. Therefore, we focused not only on speed, but also on stability during gait and investigated gait parameters.

Analysis of gait function in patients with PSP-RS showed that step length on the symptom-dominant side was significantly increased and ES was moderate. The step length on the non-symptom-dominant side was not significantly different, but increased with moderate ES. This result supports the previously reported increase in step length with training [11]. Although there was no significant improvement in the CV and SI, the CV of the gait cycle and stance phase on the symptom-dominant side increased with moderate ES. Gait dysfunction in PSP has previously been reported to be characterized by a decreased gait speed, narrow stride length, wide base, increased CV of gait cycle duration, and asymmetry [33,47,48,49]. Further, studies reported that the CV of stride length/speed during circular walking is significantly higher in PSP patients than during straight-ahead walking, and worse gait parameters during circular walking are associated with an increased risk of falls [50]. Our results showed increased stride length, but not increased CV, indicating no improvement in gait stability. Therefore, to improve the gait stability of PSP-RS patients, gait training focusing on improving the control of gait speed and balance when turning around, adjustment of gait rhythm (phase reset), and asymmetry, along with balance training for postural stability, should be introduced. In contrast, the comparison of gait function pre- and post-intervention in the PSP-PGF group revealed different results compared to the PSP-RS group. Although there was no increase in stride length, the variability in stride length on the symptom-dominant side was significantly reduced, while the effect was moderate. The asymmetry in the stance phase also significantly improved, and the effect was moderate. In atypical PSP, there has been one report of improved gait speed, symmetry, and accuracy with boxing, step tasks, and treadmill training [20]. In the PSP-PGF group, improvements were obtained for some indicators of gait stability, but not for other parameters, such as stride length. PSP-PGF is characterized by freezing of gait, such as difficulty initiating gait and “freezing” during walking and akinesia of gait [51]. Therefore, to improve gait in PSP-PGF, it is necessary to focus on interventions to improve freezing of gait and stride length in addition to gait stability and gait speed. Therefore, it is necessary to develop rehabilitation interventions for each subtype to improve gait disturbances.

The present study showed that a 2-week short-term rehabilitation intervention improves balance function in both PSP-RS and -PGF. However, data on the effects of PSP on posture and gait stability in PSP are insufficient, and further studies are required. Injuries caused by repeated falls are a significant problem in patients with PSP. Future research should include a large prospective study on PSP falls as an outcome.

This study had several limitations. Firstly, this was a retrospective study conducted at a single facility with a small sample size, lack of a control group, and absence of follow-up. In addition, the sample size was small, which reduced statistical power. The size of the PSP-PGF group was minimal. Furthermore, there were significant differences in the number of patients between the PSP-PGF and PSP-RS groups, which might have influenced the significance of the group comparisons.

Further prospective multicenter studies with larger sample sizes, randomized controlled trial settings, and follow-up of long-term rehabilitation are required to validate and support our findings. This study mainly focused on the physical and gait functions of patients with PSP, but the impact of therapeutic intervention on patients’ QoL remains unknown. In the future, the effect of rehabilitation on the QoL of patients with PSP based on PSP-QoL [52] or EQ-5D [53] should be investigated. Finally, we did not collect patient feedback on the intervention practicality or benefits, but their feedback would enrich the intervention program and its clinical applicability.

## 5. Conclusions

In this study, we showed that a 2-week short-term rehabilitation intervention program exerted beneficial effects on balance in patients with PSP-RS and PSP-PGF. In addition, we found that the stride length on the symptom-dominant side increased in the PSP-RS group, and that the asymmetry and variability of the stride length on the symptom-dominant side improved in the PSP-PGF group. Because the effectiveness of rehabilitation interventions varies according to clinical subtype, rehabilitation strategies should be specifically developed for each subtype in the future. Although these results are preliminary and require further validation, this is nevertheless a valuable report that demonstrates the effectiveness of rehabilitation for PSP.

## Figures and Tables

**Table 1:** Demographic and clinical characteristics of patients with PSP-RS and PSP-PGF.

		PSP-RS	PSP-PGF	*p*-Value
Numbers		25	8	-
Age (years) †		71.8 ± 5.8	71.0 ± 6.5	0.789
Sex (Male/Female) #		16/9	4/4	0.578
Disease duration (months) †		41.3 ± 28.5	77.3 ± 35.4	0.006 *
MMSE		26.7 ± 2.5	25.6 ± 5.3	0.821
FAB		12.9 ± 2.8	13.3 ± 4.0	0.522
Symptom-Dominant side (Right/Left) #		10/5	5/3	0.352
Body height (cm) †		163.6 ± 8.1	159.6 ± 8.0	0.220
Body weight (kg) †		56.6 ± 9.9	55.2 ± 7.0	0.821
BMI		21.1 ± 3.0	21.7 ± 2.3	0.522
mRS §		3 [3.0–4.0]	4 [2.3–4.0]	0.665
PSPRS §	I History	7 [5.5–9.0]	8 [6.0–9.0]	0.624
	II Mentation	2 [1.0–4.0]	2 [1.0–3.0]	1.000
	III Bulbar	3 [2.0–4.0]	3 [2.0–5.0]	0.324
	IV Ocular Motor	8 [3.0–10.0]	6 [3.0–8.0]	0.346
	V Limb motor	3 [3.0–5.0]	4 [3.0–5.0]	0.242
	VI Midline/Gait	8 [6.0–10.0]	10 [9.0–10.0]	0.135
	Total	29 [23.5–37.0]	32 [29.0–35.0]	0.420

RS, Richardson’s syndrome; PGF, progressive gait freezing; MMSE, Mini-Mental State Examination; FAB, frontal assessment battery; BMI, body mass index; mRS, modified Rankin scale; PSPRS, PSP rating scale. Data are shown by †: mean ± standard deviation, #: numbers, and §: median [interquartile range]. * indicates *p* < 0.05.

**Table 2:** Comparisons and ES of balance and motor function pre (0 W) and post (2 W) rehabilitation in patients with PSP-RS and PSP-PGF.

		PSP-RS *n* = 25				PSP-PGF *n* = 8			
		0 W	2 W	*p*-Value	ES (Degree)	0 W	2 W	*p*-Value	ES (Degree)
Pull test, score		2.1 ± 0.8 (1.5–3)	1.9 ± 0.8 (0–3)	0.366	0.18 (Small)	2.5 ± 0.5 (2–3)	2.5 ± 0.5 (2–3)	1.000	0.00 (No effect)
BBS, score		40.4 ± 8.7 (19–52)	43.9 ± 6.7 (29–54)	0.002 *	0.57 (Large)	43.0 ± 7.7 (32–53)	48.1 ± 5.4 (37–54)	0.002 *	0.48 (Moderate)
TUG, sec		12.8 ± 3.6 (8.0–20.2)	12.8 ± 3.3 (7.7–19.3)	0.996	0.00 (No effect)	15.3 ± 7.0 (6.1–25.6)	12.1 ± 3.2 (6.4–15.8)	0.093	0.34 (Moderate)
10 MWT, m/min		73.1 ± 17.5 (44.9–113.0)	68.5 ± 14.1 (42.1–92.7)	0.096	0.33 (Moderate)	72.2 ± 16.0 (49.4–102.2)	74.3 ± 16.9 (50.1–53.0)	0.674	0.08 (Trivial)
2 MWT, m		113.4 ± 24.6 (75–168)	111.9 ± 20.9 (75–162.5)	0.716	0.08 (Trivial)	109.8 ± 37.5 (60–190)	123.6 ± 24.9 (104–180)	0.207	0.25 (Small)
Knee Extensor Strength–weight ratio, kgf/kg	SD	35.6 ± 9.1 (9.6–51.3)	35.3 ± 9.8 (9.8–51.3)	0.796	0.05 (Trivial)	33.9 ± 13.5 (17.7–58.5)	33.9 ± 11.6 (22.3–52.1)	0.889	0.03 (Trivial)
	SND	36.0 ± 9.4 (20.0–54.0)	38.5 ± 10.0 (20.2–58.0)	0.093	0.57 (Large)	34.1 ± 10.7 (22.5–52.7)	35.9 ± 11.6 (19.9–54.8)	0.208	0.25 (Small)

RS, Richardson’s syndrome; PGF, progressive gait freezing; ES, effect size; BBS, Berg Balance Scale; TUG: Timed up and go test; 10 MWT: 10 m Walk Test; 2 MWT: 2 min walking test; SD, symptom-dominant side; SND, symptom-free side. Data were reported as mean ± standard deviation (minimum − maximum). * indicates *p* < 0.05.

**Table 3:** Comparisons and ES of gait function pre (0 W) and post (2 W) rehabilitation in patients with PSP-RS and PSP-PGF.

		PSP-RS				PSP-PGF			
		0 W	2 W	*p*-Value	ES (Degree)	0 W	2 W	*p*-Value	ES (Degree)
Gait parameters									
Gait cycle, s	SD	1.11 ± 0.11	1.13 ± 0.11	0.311	0.21 (Small)	1.10 ± 0.18	1.13 ± 0.20	0.235	0.24 (Small)
	SND	1.12 ± 0.12	1.13 ± 0.14	0.450	0.15 (Small)	1.09 ± 0.16	1.14 ± 0.19	0.160	0.28 (Small)
Stance phase, s	SD	0.70 ± 0.09	0.71 ± 0.09	0.552	0.12 (Small)	0.70 ± 0.14	0.72 ± 0.15	0.161	0.28 (Small)
	SND	0.71 ± 0.09	0.72 ± 0.09	0.881	0.03 (Trivial)	0.70 ± 0.11	0.72 ± 0.15	0.483	0.14 (Small)
Swing phase, s	SD	0.42 ± 0.05	0.43 ± 0.05	0.308	0.21 (Small)	0.41 ± 0.05	0.41 ± 0.06	0.481	0.14 (Small)
	SND	0.42 ± 0.04	0.42 ± 0.05	0.227	0.25 (Small)	0.40 ± 0.08	0.42 ± 0.06	0.262	0.22 (Small)
Step length, cm	SD	42.5 ± 7.8	45.3 ± 6.1	0.046 *	0.39 (Moderate)	43.9 ± 8.7	43.6 ± 7.7	0.889	0.03 (Trivial)
	SND	42.7 ± 8.6	45.5 ± 9.1	0.053	0.38 (Moderate)	42.2 ± 11.5	44.9 ± 7.0	0.293	0.21 (Small)
Step width, cm	SD	10.5 ± 3.8	10.8 ± 3.3	0.465	0.15 (Small)	9.7 ± 3.8	9.4 ± 3.6	0.327	0.20 (Small)
	SND	10.5 ± 3.7	10.8 ± 3.4	0.468	0.15 (Small)	9.4 ± 3.7	9.7 ± 3.8	0.612	0.10 (Small)
CV of gait cycle	SD	4.4 ± 2.5	5.2 ± 2.3	0.097	0.33 (Moderate)	4.4 ± 3.5	4.2 ± 3.1	0.866	0.03 (Trivial)
	SND	4.6 ± 2.4	4.0 ± 2.3	0.265	0.23 (Small)	4.4 ± 3.1	4.5 ± 2.8	0.575	0.28 (Small)
CV of stance phase	SD	5.5 ± 2.7	6.7 ± 3.7	0.079	0.35 (Moderate)	5.2 ± 3.2	6.3 ± 3.2	0.327	0.20 (Small)
	SND	6.6 ± 2.3	6.3 ± 2.7	0.376	0.18 (Small)	8.4 ± 7.3	6.3 ± 3.2	0.575	0.11 (Small)
CV of swing phase	SD	6.7 ± 4.6	6.4 ± 3.5	0.700	0.08 (Trivial)	7.7 ± 4.0	5.8 ± 2.1	0.327	0.20 (Small)
	SND	6.6 ± 3.9	6.6 ± 4.7	0.784	0.06 (Trivial)	8.2 ± 5.0	4.9 ± 2.3	0.161	0.28 (Small)
CV of step length	SD	9.3 ± 7.4	8.3 ± 4.2	0.407	0.17 (Small)	8.8 ± 3.4	5.3 ± 2.2	0.036 *	0.42 (Moderate)
	SND	8.5 ± 4.1	8.6 ± 4.8	0.915	0.02 (Trivial)	8.9 ± 4.1	6.3 ± 3.2	0.068	0.37 (Moderate)
CV of step width	SD	27.4± 18.3	25.7 ± 12.9	0.920	0.02 (Trivial)	31.6 ± 25.6	31.6 ± 16.0	0.674	0.08 (Trivial)
	SND	26.9 ± 16.6	24.9 ±15.8	0.424	0.16 (Small)	27.6 ± 15.7	23.0 ± 11.4	0.327	0.20 (Small)
Symmetry Index									
Gait cycle		1.8 ± 1.5	1.7 ± 1.7	0.533	0.13 (Small)	1.6 ± 1.5	1.6 ± 1.8	0.889	0.03 (Trivial)
Stance phase		4.5 ± 3.5	4.4 ± 3.1	0.903	0.02 (Trivial)	6.1 ± 4.2	2.7 ± 2.2	0.025 *	0.45 (Moderate)
Swing phase		7.4 ± 5.4	7.6 ± 5.2	0.864	0.03 (Trivial)	6.2 ± 4.3	2.7 ± 2.4	0.161	0.28 (Small)
Step length		10.9 ± 7.8	10.3 ± 8.7	0.715	0.07 (Trivial)	12.4 ± 10.4	6.6 ± 6.7	0.161	0.28 (Small)
Step width		14.8 ± 13.8	13.0 ± 8.7	0.568	0.11 (Small)	18.1 ± 15.4	17.0 ± 8.1	1.000	0.00 No Effect

RS, Richardson’s syndrome; PGF, progressive gait freezing; ES, effect size; CV, coefficient of variation; SD, symptom-dominant side; SND, symptom non-dominant side. Data are reported as mean ± standard deviation (minimum − maximum). * Indicates *p* < 0.05.

## Data Availability

The original contributions presented in the study are included in the article, and further inquiries can be directed to the corresponding author.

## Appendix A

**Table A1:** Overview of the multiple therapeutic exercise program.

Program	Detailed Description of Programs
Range of motion exercise and stretching	Stretching and joint range-of-motion exercises are performed passively on the restricted joints and muscles, assisted by the therapist, to improve postural alignment.
Muscle strength training	Loading by the patient’s own weight or by the hand of the therapist on the limbs, neck, and trunk. Focuses on core training and performing leg raises, bridges, crunch, squats, heel and toe raises, front lunges, side lunges, etc.
Balance training	Under the guidance of the therapist, balance training is performed to improve postural stability and control. Depending on the patient’s motor function, the degree of difficulty can be adjusted using a balance cushion or performing with closed eyes.
	Moving the center of gravity back, forth, left, or right in sitting position; Quadruped leg lift in all-fours position; Holding upper limb elevation or rotating the trunk in kneeling or half-kneeling positions; Holding standing position on a stable or an unstable surface in the opened leg standing, closed leg standing, tandem standing, or one leg standing positions. Adjusting the level of difficulty using a balance pad/disc or performing with closed eyes. Reaching back, forth, left, or right; Stepping back, forth, left, or right; Turning clockwise or anticlockwise.
Gait training	Gait training focuses on balancing gait so that the patient maintains postural stability while walking. Side walking; Tandem walking; Cross step walking; Backward walking; Treadmill training; Shuttle walking; Sudden starts and stops; Walking and turning around. In addition, training on how to walk safely with a cane or walking aids is provided.
Activities of daily living training	Transfers: getting up from a bed, rising up from a chair, transferring to a chair or bed, and sitting down in the chair; Toilet use: getting to and from the toilet, turning in the toilet, and cleaning oneself; Bathing: transferring in the bathroom; straddling the bath tub; washing one’s face, hair, and body; Dressing: Putting on and taking off upper and lower garments; Mobility: walking indoors and outdoors; Stairs: going up and down stairs.
